# Maternal One-Carbon Supplement Reduced the Risk of Non-Alcoholic Fatty Liver Disease in Male Offspring

**DOI:** 10.3390/nu14122545

**Published:** 2022-06-19

**Authors:** Hui Peng, Huiting Xu, Jie Wu, Jiangyuan Li, Xian Wang, Zhimin Liu, Minjee Kim, Minsun S. Jeon, Ke K. Zhang, Linglin Xie

**Affiliations:** 1 Department of Nutrition, Texas A&M University, College Station, TX 77840, USA; denice1010@163.com (H.P.); wangxian18@tamu.edu (X.W.); chris903@163.com (Z.L.); 2 Department of Pathology, University of North Dakota, Grand Forks, ND 58202, USA; annexu333@126.com; 3 Center for Epigenetics & Disease Prevention, Institute of Biosciences & Technology, College of Medicine, Texas A&M University, Houston, TX 77840, USA; jiewu2014@whu.edu.cn (J.W.); minsunjeon@tamu.edu (M.S.J.); 4 Department of Statistics, Texas A&M University, College Station, TX 77840, USA; jiangyuanli@stat.tamu.edu (J.L.); minjeekim0804@tamu.edu (M.K.)

**Keywords:** non-alcoholic fatty liver disease (NAFLD), pregnancy, offspring, high-fat diet, one-carbon supplement, epigenetics, DNA methylation

## Abstract

Recent studies have suggested that prevention of obesity and non-alcoholic fatty liver disease (NAFLD) should start with maternal dietary management. We previously reported disrupted methionine cycle, associated with NAFLD, in male offspring liver due to maternal high-fat (HF) diet, thus we hypothesize that maternal one-carbon supplement may reduce the risk of NAFLD in offspring via the normalizing methionine cycle. To test it, female mice (F0) were exposed to either a maternal normal-fat diet (NF group) a maternal HF diet (HF group), or a maternal methyl donor supplement (H1S or H2S group) during gestation and lactation. The offspring male mice (F1) were exposed to a postweaning HF diet to promote NAFLD. While the HF offspring displayed obesity, glucose intolerance and hepatic steatosis, the H1S and H2S offspring avoided hepatic steatosis. This phenotype was associated with the normalization of the methionine cycle and the restoration of L-carnitine and AMPK activity. Furthermore, maternal HF diet induced epigenetic regulation of important genes involved in fatty acid oxidation and oxidative phosphorylation via DNA methylation modifications, which were recovered by maternal one-carbon supplementation. Our study provides evidence that maternal one-carbon supplement can reverse/block the adverse effects of maternal HF diet on promoting offspring NAFLD, suggesting a potential nutritional strategy that is administered to mothers to prevent NAFLD in the offspring.

## 1. Introduction

Nonalcoholic fatty liver disease (NAFLD) is closely associated with obesity, type 2 diabetes, hypertension and hyperlipidemia, and is the liver manifestation of metabolic syndrome, which has a significant impact on global health [1]. 2015, 25% of the global adult population was affected by NAFLD, with regional differences [2]. East Asia has the highest case numbers, followed by South Asia. However, the highest prevalence of NAFLD was observed in North Africa and the Middle East, while Western Europe had the greatest increase [3]. Since the incidence and severity of obesity is increasing at an alarming rate, all classifications of NAFLD are trending higher in prevalence [4]. The longitudinal risk of NAFLD is now of greater focus in children and adolescents, where rapid weight gain during school years is directly associated with the histological characteristics of NAFLD in adulthood [4].

It is now well established that in utero and early life exposure to under- or over-nutrition can disrupt normal growth and development, and this thus changes the offspring phenotype to one that is prone to future diseases [5,6,7,8,9,10,11,12]. The risk of childhood obesity is higher in kids whose mothers are obese prior to pregnancy [13]. Importantly, the recent concept of “the first 1000 days”, defined by the period from conception to age of two, highlights the best time for obesity prevention [14]. Therefore, obesity prevention should emphasize preconception and pregnancy care.

Previous work has demonstrated that maternal dietary restriction or the supplementation of a methyl-donor both altered lipid metabolism and body fat composition in offspring [15,16,17], suggesting a potential link between maternal HF diet and the offspring obesity and NAFLD via the level of methyl donor. Impaired folate metabolism (methyl-trap) has also been reported in offspring exposed to maternal high-fat (HF) diets in different animal models [18,19,20,21]. In our previous study, we reported that the maternal HF diet disrupts methionine cycle in the male offspring liver, which further inhibits the mitochondrial β-oxidation of long-chain fatty acids via reduced L-carnitine production [21]. Thus, we will test a hypothesis that maternal methyl donor supplementation will block the adverse effect of maternal HF diet on promoting offspring obesity and NAFLD. In this study, female pregnant mice exposed to overnutrition induced by an HF diet will be treated with two different types of maternal one-carbon supplement, the obesity and NAFLD phenotypes of male offspring will be examined and the underlying molecular and epigenetic mechanisms will be investigated.

## 2. Materials and Methods

### 2.1. Experimental Design 

Mixed background (B6/129/SvEv) four-week-old female mice were used in this study and were fed either a high fat diet (60% kcal from fat) or a normal fat diet (10% kcal from fat, the NF group) for 12 weeks. One third of the female mice on the HF diet continued to be fed an HF diet throughout gestation and lactation (the HF group). During gestation and lactation, the rest of the female mice on the HF diet were treated with the HF diet combined with either a methionine supplement (the H1S group) or a complete maternal one-carbon supplement (the H2S group) (Table 1).

After weaning, a high fat diet was fed to male offspring mice from all of the groups for 12 weeks to promote weight gain prior to sacrifice. A separate group, birthed from the breeders who adhered to the NF diet, were continuously fed the NF diet for 12 weeks and were utilized as a reference control group (the REF group). Mouse experiments were completed according to a protocol that was reviewed and approved by the Institutional Animal Care and Use Committee of the University of North Dakota and Texas A&M University, in compliance with the USA Public Health Service Policy on Humane Care and Use of Laboratory Animals. Approval Code: 2019-0309; Approval Date: 19 September 2019.

### 2.2. Diet Composition

The diets were purchased from Research Diets, LLC (New Brunswick, NJ, USA). The NF (normal fat) diet (Cat# D12450B) had an energy density of 3.771 kcal/g (10% fat energy, 70% carbohydrate energy, and 20% protein energy). The HF (high fat) diet (Cat# D12492) had an energy density of 5.157 kcal/g (60% fat energy, 20% carbohydrate energy, and 20% protein energy). The fat source was composed of 92% lard and 8% soybean oil. The H1S + HF diet added a methionine supplement (L-methionine 7.5 gm/kg) to the HF diet, and the H2S + HF diet added a complete maternal one-carbon supplement (15 g/kg choline Chloride, 15 g/kg betaine (anhydrous), 15 mg/kg folic acid, 1.5 mg/kg vitamin B12, 7.5 g/kg L-methionine, and 150 mg/kg zinc from ZnSO_4_·7H_2_O) to the HF diet.

### 2.3. Intraperitoneal Injected Glucose Tolerance Test (IPGTT)

At the end of week 12, offspring mice from each experimental group were fasted overnight for 12 h and were subjected to an IPGTT early the next morning. Glucose tolerance tests were conducted with 20% D-glucose in 0.9% saline, and the final concentration of the administered dose was 2.0 g/kg of body weight.

### 2.4. UPLC-MS/MS

The liver content of methionine, SAM, SAH, L-Carnitine and Creatine was measured by LC-MS analyses as described in our previous reports [20]. The following chemicals were used as standards: L-methionine-d3, acetyl-d3-L-carnitine∙HCl, (RS)-S-adenosyl-L-methionine-d3 (S-methyl-d3) tetra (p-toluenesulfonate) salt, and creatine-d3 H_2_O (methyl-d3) [CDN isotopes, Quebec, QC, Canada]; and S-Adenosylhomocysteine-d4 [Cayman Chemical, Quebec, QC, Canada].

### 2.5. SAM-Dependent Methyltransferase Activity

SAM-dependent methyltransferases were measured by an enzyme-coupled assay using the Methyltransferase Colorimetric Assay Kit (Cayman Chemical, Ann Arbor, MI, USA), according to the manufacturer’s instructions.

### 2.6. DNA Methyltransferase Activity

Total DNMT activity was measured using the EpiQuik DNA Methyltransferase (DNMT) Activity/Inhibition Assay Kit (Epigentek, Brooklyn, NY, USA), according to the manufacturer’s instructions. The ratio or amount of methylated DNA, which is proportional to enzyme activity, was calorimetrically quantified through an ELISA-like reaction.

### 2.7. Genomic DNA Methylation Level

Genomic DNA was extracted using a Quick-DNATM Universal Kit (Zymo Research, Irvine, CA, USA) according to the manufacturer’s instructions. The DNA methylation level was determined in triplicate with a NanoDrop ND-1000 spectrophotometer (Thermo Fisher Scientific, Waltham, MA, USA). Following the manufacturer’s instructions for the MethylFlash Methylated DNA 5-mC Quantification Kit (Colorimetric) (Epigentek, Brooklyn, NY, USA), global DNA methylation was quantified. The methylated fraction of DNA was detected using capture and detection antibodies provided with the kit and then quantified through an ELISA-like reaction by reading the absorbance in a microplate spectrophotometer at 450 nm.

### 2.8. Antibodies

An antibody against PPAR-α was purchased from Millipore Sigma (Burlington, MA, USA). Antibodies against AMPK-α, Phospho-AMPK-α (Thr172) and GAPDH were purchased from Cell Signaling Technology (Danvers, MA, USA).

### 2.9. Real-Time PCR (RT-PCR)

Total RNA was extracted from liver tissue with TRIzol reagent (Thermo Fisher Scientific, Waltham, MA, USA), and the RNA concentration was determined in triplicate with a NanoDrop ND-1000 spectrophotometer (Thermo Fisher Scientific, Waltham, MA, USA). Total mRNA (1 µg) was amplified and reversed transcribed with the ReadyScript^®^cDNA Synthesis Mix (Sigma-Aldrich, Burlington, MA, USA), and all reactions were performed in triplicate using a Bio-Rad real-time PCR machine with CFX Manager 3.1 software. The ΔCT values were used for statistical analysis.

### 2.10. Whole Genome Bisulfite Sequencing (WGBS)

Genomic DNA of liver was extracted by Quick-DNA Universal Kit (ZYMO Research, USA), and was broken into 400–500 bp fragments using sonication(Qsonica q700). End repair and adaptor ligation were performed by the following 2 kits: NEBNext^®^ Ultra™ II DNA Library Prep with Sample Purification Beads (NEB, USA) and NEBNext^®^ Multiplex Oligos for Illumina^®^ (Methylated Adaptor, Index Primers Set 1) (NEB, USA). Adaptor-ligated DNA was cleaned up without size selection. Bisulfite conversion was performed by MethylCode™ Bisulfite Conversion Kit (Invitrogen, USA). PCR amplification of adaptor-ligated and bisulfite converted DNA were performed by KAPA HiFi Hotstart Uracil + ReadyMix PCR Kit (KAPA Biosystems, USA). The 1.8pM library and 20pM PhiX control (Illumina, USA) were prepared following the NextSeq System Denature and Dilute Libraries Guide using NextSeq 500/550 v2 Kit (high output 150 cycle) (Illumina, USA). WGBS was performed in the Center for Epigenetics & Disease Prevention, Institute of Biosciences & Technology, Texas A&M University (Houston, TX, USA). WGBS data is now accessible at the BioProject database (BioProject ID: PRJNA777555).

### 2.11. Statistical Analysis

Measurements for single time points were analyzed by Fisher’s least significant difference test to allow multiple comparisons of differences between the groups REF, NF, HF, H1S, and H2S. Fisher’s least significant difference test was performed by first performing a one-way analysis of variance for all treatment groups. For longitudinal data, such as body weight, a linear mixed model was used for the analysis of repeated measures, with each individual mouse as a random effect. A *p* value less than 0.05 was considered to be significant, while a *p* value less than 0.1 was considered to be marginally significant. All analyses were performed using SAS JMP software (SAS Institute Inc., Cary, NC, USA).

To detect differential DNA methylation and identify differentially methylated CpG sites, Fisher’s exact test was performed between the methylation status and the two treatment groups, HF and H2S. A *p* value less than 0.05 was considered significant. An odds ratio was calculated to evaluate the pattern of methylation and the direction of CpG methylation change. Specifically, whether the HF diet led to an increase or decrease in hypermethylation or hypomethylation was examined. If the 95% confidence interval for the odds ratio was greater than 1, it was concluded that there was a decrease in methylation from HF to H2S. Furthermore, we wanted to test whether there were CpG sites that were recovered by the H2S diet. If the absolute methylation difference (%) between HF and NF for differentially methylated regions (DMRs) was greater than or equal to 10, the site was considered to be significantly methylated. If the absolute methylation difference for the significantly methylated site in the HF group transitioned to values between −10 and 10 in the H2S group, it was considered as a recovery case. All analyses were performed using R Statistical Software (Foundation for Statistical Computing, Vienna, Austria).

## 3. Results

### 3.1. Maternal One-Carbon Supplementation Allowed Offspring to Avoid NAFLD Promoted by a Maternal HF Diet but Not Obesity and Glucose Intolerance

**Figure 1:**
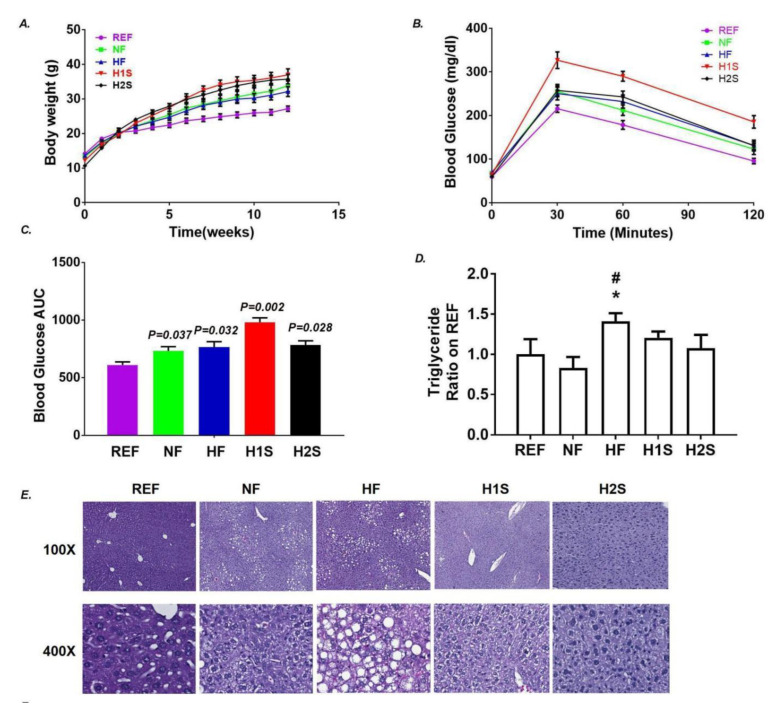
Maternal one-carbon supplementation allowed offspring to avoid NAFLD promoted by a maternal HF diet but not obesity and glucose intolerance. (**A**) Body weights of male offspring were recorded weekly after weaning for 12 weeks. (**B**) IPGTT was measured at the end of week 12 in male offspring. (**C**) The areas under the curve (AUCs) were calculated for the IPGTT results in male offspring. (**D**) Serum triglyceride was detected in male offspring. (**E**) H&E staining was performed on the liver tissue of male offspring. Data are presented as the mean ± SE, *n* = 15. * indicates *p* < 0.05 vs. the NF group. # indicates *p* < 0.05 vs. the REF group.

We observed significantly more body weight gain in the NF and HF offspring than the REF offspring, as expected. However, we surprisingly observed no difference in the body weight of the HF/NF offspring and the offspring exposed to a maternal methionine supplement (H1S diet) or a complete maternal one-carbon supplement (H2S) (Figure 1A). Similar to the NF and HF offspring, both the H1S and the H2S offspring were glucose intolerant by the end of week-12 with HFD. However, while HF offspring had significantly higher serum TG contents, the H1S and H2S offspring presented normal serum TG (Figure 1D). Hepatic steatosis and the largely distributed ballooning cells, observed in the HF offspring, was not present in the NF, H1S and H2S offspring (Figure 1E).

### 3.2. Maternal One-Carbon Supplementation Corrected and Overloaded the Disrupted Methionine Cycle by Maternal HF Diet

**Figure 2:**
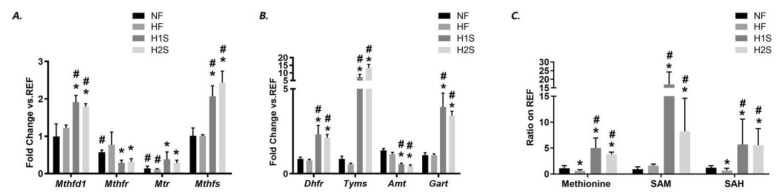
Maternal one-carbon supplementation corrected and overloaded the disrupted methionine cycle by maternal HF diet. (**A**) Gene expression levels of Mthfd1, Mthfr, Mtr, and Mthfs were measured by real-time PCR in the male offspring livers. (**B**) Gene expression levels of Dhfr, Tyms, Amt, and Gart were measured by real-time PCR in the male offspring livers. (**C**) Methionine, SAM and SAH contents were detected via high-resolution UPLC-MS/MS. Data are presented as the mean ± SE, *n* = 5. * indicates *p* < 0.05 vs. the NF group. # indicates *p* < 0.05 vs. the REF group.

With the methionine or complete one-carbon supplement, we wonder if the expression of genes involved in folic acid metabolism was affected. Both the H1S and the H2S diets significantly changed the offspring expression levels of many key genes involved in folic acid metabolism. These include the genes encoding proteins that positively regulate folic acid metabolism: Mthfd1, Mthfr, Mtr and Mthfs (Figure 2A), and those that negatively regulate it: Dhfr, Tyms, Amt and Gart (Figure 2B). Compared to that, the HF diet significantly decreased Mtr expression, and the H1S and H2S diets significantly increased expression of Mthfd1, Mthfs, Dhfr, Tyms and Gart, but decreased Mthfr and Amt expression. For Mtr encoding Methionine synthase (MS), which is responsible for the regeneration of methionine from homocysteine, H1S and H2S diets partially rescued its depressed expression by the maternal HF diet, although its level was still lower than REF offspring. For the other overexpressed genes, their expression levels were even higher than the NF offspring.

We further measured the hepatic content of important intermediate chemicals of the methionine cycle including methionine, SAM and SAH. Consistent with our previous report [20], the HF diet significantly reduced hepatic methionine and SAH content. However, the H1S and the H2S offspring had dramatically increased levels of hepatic methionine, which was about four- to five-fold of the reference level. Consistently, the H1S and H2S offspring dramatically increased the hepatic SAM and SAH content compared with the REF, NF and HF offspring (Figure 2C).

### 3.3. Maternal One-Carbon Supplementation Overactivated the SAM-Transferase Activity but Did Not Correct the Genomic DNA Hypermethylation Caused by a Maternal HF Diet

**Figure 3:**
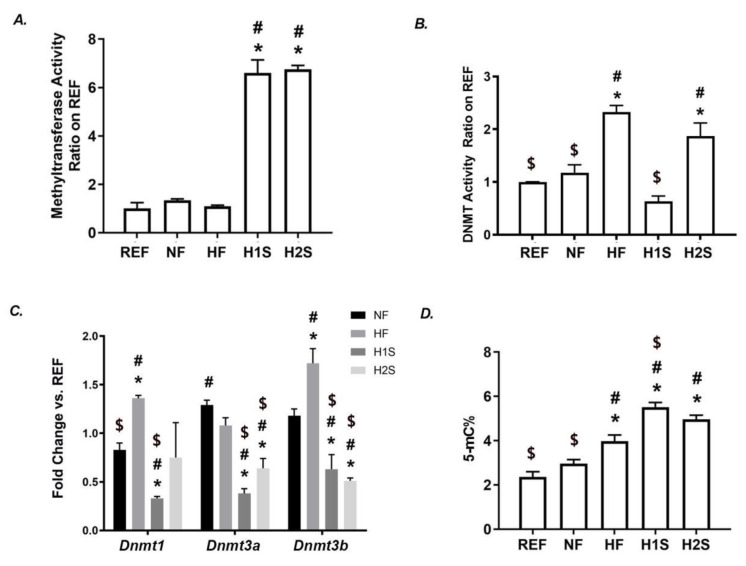
Maternal one-carbon supplementation overactivated the SAM-transferase activity but did not correct the genomic DNA hypermethylation caused by a maternal HF diet. (**A**) The enzymatic activity of SAM was detected in male offspring. (**B**) The enzymatic activity of DNMT was detected in male offspring. (**C**) Hepatic gene expression levels of Dnmt1, Dnmt3a and Dnmt3b in male offspring were measured by real-time PCR. (**D**) Genomic DNA methylation levels were detected in the male offspring livers. Data are presented as the mean ± SE, *n* = 5. * indicates *p* < 0.05 vs. NF group. # indicates *p* < 0.05 vs. the REF group. $ indicates *p* < 0.05 vs. the HF group.

Consistent with the enhanced SAM and SAH levels, SAM-transferase overactivity was observed in the H1S and the H2S offspring compared with the REF, NF and HF offspring (Figure 3A). Interestingly, while the HF diet significantly overactivated DNMT in offspring, the H1S diet completely eliminated such an effect, while the H2S diet did not (Figure 3B). The gene expression level of Dnmt1, Dnmt3a and Dnmt3b was further assessed by RT-PCR. The significantly increased expression of Dnmt1 and Dnmt3b were observed in HF liver compared to the REF or NF offspring. However, the H1S diet resulted in the decreased expression of all three genes compared to either REF or NF offspring. Notably, the H2S diet only corrected the lower expression level of Dnmt1 caused by the maternal HF diet. The H2S diet also over-reversed the higher expression of Dnmt3a and Dnmt3b to levels even lower than the NF or REF offspring (Figure 3C). Interestingly, similar to the HF offspring, the H1S and the H2S offspring still displayed hypermethylation of genomic DNA and its level of H1S was even higher than that of the HF offspring (*p* < 0.05, Figure 3D).

### 3.4. Maternal One-Carbon Supplementation Promoted Methyl Group Delivery to L-Carnitine and Normalized PPARα Expression and the AMPK Signaling Activation

**Figure 4:**
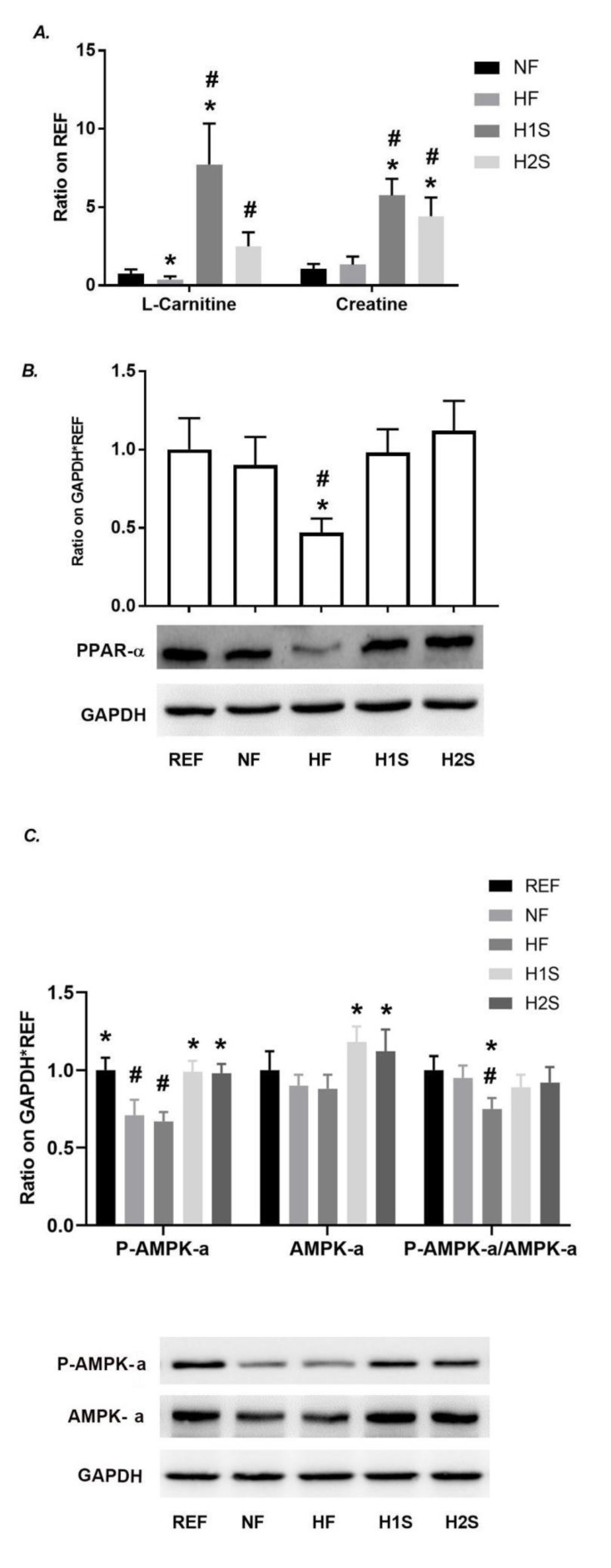
Maternal one-carbon supplementation promoted methyl group delivery to L-carnitine and normalized PPARα expression and the AMPK signaling activation. (**A**) L-carnitine and creatine content was detected via high-resolution UPLC-MS/MS. (**B**) PPAR-α expression was detected by western blots in male offspring. (**C**) AMPK-α and phospho-AMPK-α (Thr172) expression were detected by western blots in male offspring. Relative amounts were expressed as the ratio of Phospho- AMPK-α (Thr172)/AMPK-α. Data are presented as the mean ± SE, *n* = 5. * indicates *p* < 0.05 vs. the NF group. # indicates *p* < 0.05 vs. the REF group.

Consistent with the previous report, we repeatedly observed the decreased hepatic content of L-carnitine, a methylated product of guanidinoacetate acid, in HF offspring (Figure 4A). Dramatically increased levels of hepatic L-carnitine and creatinine were observed in the H1S and H2S offspring compared with those in the REF, NF or HF offspring (Figure 4A). H1S and H2S also increased the level of creatine up to six-fold of the REF offspring, another product of SAM-transferase catalyzed product (Figure 4A). These results suggested an overloading of the methionine cycle pushed by maternal H1S and H2S diets.

Considering that the main function of carnitine is to transfer the long-chain fatty acids to mitochondria for subsequent β-oxidation, we tested to see whether the level change of L-carnitine was associated with the level of fatty acid oxidation. Consistent with this change, the reduced PPAR-α expression level caused by the HF diet was recovered in the H1S and H2S offspring (Figure 4B). Moreover, the H1S and H2S diets blocked the effects of the NF and HF diets on the inhibition of AMPK signaling in the liver. The AMPK and the p-AMPK expression of the H1S and H2S offspring livers were similar to those in the REF offspring liver. The levels of p-AMPK-α/AMPK-α was significantly lower in HF offspring than NF or REF offspring. However, these ratios in H1S and H2S offspring were not different from REF or NF offspring (Figure 4C).

### 3.5. Genes Involved in Lipid Metabolism and Oxidative Phosphorylation, Whose Hepatic Expression Was Associated with DNA Methylation Modification, Were Identified in the Offspring Liver

**Figure 5:**
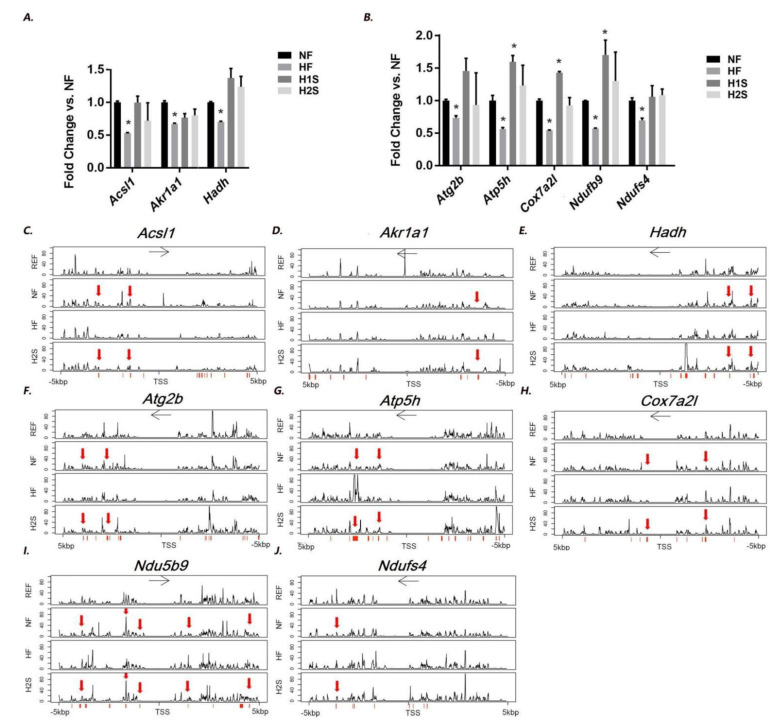
Genes involved in lipid metabolism and oxidative phosphorylation, whose hepatic expression was associated with DNA methylation modification, were identified in the offspring liver. (**A**) Gene expression levels of Acsl1, Akr1a1, and Hadh were measured by real-time PCR in the male offspring livers. Data are presented as the mean ± SE, *n* = 5. * indicates *p* < 0.05 vs. NF group. (**B**) Gene expression levels of Atg2b, Atp5h, Cox7a2l, Ndufb9 and Ndufs4 were measured by real-time PCR in the male offspring livers. (**C**–**J**) DNA methylation patterns of a 10 kbp genomic region extending from 5 kbp upstream of the TSS to 5 kbp downstream of the TSS were analysed by WGBS. The *y*-axis indicates the percentage of methylation at each DNA nucleotide. Only those DNA methylation changes of more than 20% were considered potential methylation pattern changes. The *x*-axis indicates the location of each DNA nucleotide around the TSS. Typical DMRs induced by maternal HF diet but recovered by H2S diet are labeled by red arrow. Modified CpG loci are labeled with short red lines on the *x*-axis.

We have previously reported differentiated genes (DEGs) between the HF group and the NF group by RNA-seq analysis [21]. Thus, by RT-PCR we validated the expression change of DEGs involved in two KEGG signaling pathways: the NAFLD pathway and the oxidative phosphorylation pathway. These included the NAFLD-related genes including Acsl1, Akr1a1 and Hadh and oxidative phosphorylation genes including Atg2b, Atp5h, Cox7a2l, Ndufb9 and Ndufs4. It is noted that Hadh is reported to be a target of PPARα [22]. All repressed their expression in HF offspring liver (Figure 5A,B). The H2S diet normalized the expression of all these genes in the offspring livers. The H1S diet completely reversed the expression of all NAFLD-related genes and two oxidative phosphorylation genes, Atg2b and Ndufs4. The H1S diet also further enhanced the expression of Atp5h, Cox7a2l and Ndufb9 to a level even higher than the NF offspring liver.

We further asked if the gene expression changes by different maternal diets were associated with the DNA methylation changes in the offspring liver. Thus, WGBS was performed to detect the differentiated methylation region (DMR) between the HF vs. NF and H2S vs. NF. Thus, regions with a methylation difference more than 10% were screened within the 10 kbp genomic region that covers the 5 kbp upstream and 5 kbp downstream of the gene’s TSS site. The HF offspring displayed distinct DNA methylation patterns in several genomic regions of the eight genes; however DNA methylation patterns in most of the regions were not different between H2S and NF offspring (Figure 5C–J, indicated by red arrow).

The DNA methylation pattern differences were further analyzed to elucidate if the DNA methylation level changes in these regions were statistically significant (see methods). For the eight genes, we identified a total of 262 DMRs modified by the HF diet and a total of 175 DMRs modified by the H2S diet (Table 2). Among the 262 HF-diet induced DMRs, 233 were normalized (96.95%, Table 3, see detailed CpG site information in Table S1). H2S recovered the majority of methylation changes induced by HF in five genes including Acsl1 (100% recovered), Atg2b (100% recovered), Atp5h (99.31% recovered), Cox7a2l (100% recovered), Ndufb9 (93.18% recovered) and Ndufs4 (100% recovered). It is interesting that the H2S diet also resulted in additional DMRs with a dominantly hypermethylation modification of the offspring liver compared to the NF offspring (Table 3 and Table S1). For the gene Akr1a1 and Hadh, HF diet only induced 4 and 1 DMRs respectively, among which only 1 DMR was recovered by H2S. However, H2S resulted in 27 and 24 additional DMRs, suggesting an association between these DMRs and the normalized expression of Akr1a1 and Hadh. We further performed Fisher’s Exact Test to determine if the H2S diet significantly modified the DMRs compared to the HF diets. The statistical results indicated that the H2S diet significantly modified DNA methylation changes induced by the HF diet on all except for Atg2b and Ndufs4, whose DMR modification between H2S and HF was marginally significant (Table 3).

## 4. Discussion

Current recommendations from the National Institute for Health and Care Excellence (NICE) and the American Congress of Obstetricians and Gynecologists (ACOG) are that women of childbearing age should modulate their BMI to a value within the normal range before conception by engaging in lifestyle changes such as moderate increases in exercise activity or improvements in the quality of their diet [23,24]. However, healthy body weight management takes a long time. Thus, dietary interventions during pregnancy are important in optimizing the pregnancy outcomes in both the mother and children. In this study, we evaluated the impacts and mechanisms of maternal one-carbon supplements on NAFLD prevention in male offspring of a high risk, HF diet (due to maternity).

Both of the different methyl donor supplements (H1S and H2S diets) prevented offspring hepatic steatosis but not offspring obesity or glucose intolerance. These findings are consistent with previous reports that maternal high folate intake increases the risk for obesity and glucose intolerance in male offspring [25,26]. However, the two diets, whether composed of methionine only or containing other essential cofactors for methyl group delivery displayed similar effects on the prevention of offspring NAFLD, suggesting that maternal supplementation with methionine only is sufficient to prevent offspring NAFLD. In this model, we confirmed the activated methionine cycle in offspring liver by maternal methyl-donor supplement, which further replenished the methyl donor and enhanced SAM-catalyzed methyl group delivery. Although several previous studies have reported that methyl donor supplementation alleviates hepatic lipid accumulation in murine models fed with a HF diet [18,19,20,27,28,29], the beneficial effects of maternal methyl donor supplementation on offspring NAFLD has never been reported before.

Normalized PPARα level and AMPK signaling activation in the offspring liver are responsible for the lipid homeostasis in the liver with maternal one-carbon supplement. Other supporting evidence includes the normalized expression of panels of genes involved in lipid metabolism and fatty acid oxidation. This result is consistent with previous reports that one carbon supplement activates AMPK signaling in various tissues and different disease models [30,31,32,33]. Importantly, hepatic L-carnitine content was greatly increased by maternal one carbon supplement, while it was significantly decreased by maternal HF diet. As a product of methyl-group transfer and a regulator of CPT1, L-Carnitine has been used as an efficient dietary supplement in body weight control in humans due to its well-known effects on promoting fatty acid oxidation [34,35,36]. To be noted, L-carnitine is an established mitochondrial biomarker used to screen neonates for a series of genetic disorders affecting fatty acid oxidation [37]. Thus, our results provided mechanistic insights into how maternal diet through one-carbon metabolism affected the offspring L-carnitine level, which further contributes to fatty acid metabolism.

Maternal diets reprogramming lipid homeostasis in the liver could be via an epigenetic mechanism involving DNA methylation modifications. Previously, we and others reported global DNA hypermethylation in the HF offspring liver with a disrupted methionine cycle, while readjusting the maternal diet to a healthier one avoided these changes with a corrected methionine cycle in the offspring liver [21], indicating an association between methionine cycle and DNA methylation. In the current study, maternal one-carbon supplement led to overactivation of the methionine cycle, evidenced by higher levels of methionine, SAM, and methylation substrates including L-carnitine, creatine and methylated-DNA, due to overactivation of SAM methyltransferase. However, DNMT, which belongs to SAM methyltransferase, behaved in the opposite manner. A maternal HF diet significantly enhanced its enzymatic activity, which was completely reversed by maternal H1S but not by an H2S diet, suggesting that a normal enzymatic activity of DNMT requires sufficient methionine but excessive levels of other co-factors for one-carbon metabolisms may not necessarily beneficial. In the liver, it is previously reported that development of hepatic steatosis was accompanied by changes in Dnmt1 and Dnmt3a expression [38]. In our study, DNMT activity changes were inconsistent with the expression change of Dnmt genes, neither with the presence of hepatic steatosis. Moreover, maternal one-carbon donor supplementation did not correct DNA hypermethylation in the offspring liver, which is unexpected. These intriguing results with unknown molecular mechanisms suggest complicated feedback mechanisms between DNMT enzymatic efficiency and one-carbon donors and intermediate transmitters contributing to hepatic steatosis. Nevertheless, our data suggested that abundant substrates and co-enzymes fueling the one-carbon metabolism could be a major driving force of genome-wide DNA hypermethylation, and a normal level of DNMT activity is capable of handling the excessive catalytic reactions.

When specific CpG loci were evaluated, except for normalizing the HF-induced DMRs, the H2S diet also caused a total of 167 additional DMRs, although without further enhancement of the gene expression. Among these DMRs, about 90% were hypermethylated. It is possible that maternal one-carbon supplement provided excessive substrates and co-enzymes, which push forward the reaction that additional CpG loci, “of nonsense”, were methylated non-specifically. If this is true, whether the stability of EU chromosome is affected should be further investigated. However, we must also point out that the methylation modification on Akr1a1 and Hadh may not support that the additional DMRs are “of nonsense” due to that the additional DMRs might be associated with the normalized gene expression. Future studies are required to validate that these hypermethylated DMRs on Akr1a1 and Hadh are responsible for the gene expression changes.

Mechanistically, it is also possible that different maternal diets result in differential DNA methylation modifications within specific genomic regions responsible for transcription regulation. We and others have reported important DMRs associated with important genes and signaling pathways such as insulin and IGF-signaling in the offspring liver responsible for the maternal HF diet [21,39,40,41,42]. Studies also reported associations between gene expression and DNA methylation on important genes involved in lipid metabolism, e.g., Lep, Ppar-α, Scd, Slc2a4, MT-ND6 (mitochondrial gene NADH dehydrogenase 6) and PGC1-α [40,43,44,45,46]. However, evidence and validation of the expression-DNA methylation association on these specific genes contributing to hepatic steatosis are missing, resulting in an obvious limitation to these studies. Nonetheless, we reported that the maternal HF diet induced a total of 262 DMRs on eight down-regulated genes involved in NAFLD development and oxidative phosphorylation. More importantly, the H2S diet normalized about 97% of the HF-induced DMRs, associated with normalized gene expression, providing strong evidence that a maternal diet reprograms the offspring metabolism associated with DNA methylation modifications.

In summary, we provided evidence to support a potential nutritional strategy that is administered to mothers to prevent NAFLD in the offspring and that suggests new directions of future human studies to mitigate the increasing trend of NAFLD, beginning with maternal dietary interventions.

## Figures and Tables

**Table 1:** Study Design *.

	Maternal Diet		Offspring Diet
	Pre-Pregnancy	Pregnancy/Lactation	after Weaning
REF (*n* = 15)	NF	NF	NF
NF (*n* = 15)	NF	NF	HF
HF (*n* = 15)	HF	HF	HF
H1S (*n* = 15)	HF	HF + H1S	HF
H2S (*n* = 15)	HF	HF + H2S	HF

* Diet Description: REF Rodent diet: normal chow diet (4% cal. from fat); NF Rodent diet: normal fat (10% cal. from fat); HF Rodent diet: high fat (60% cal. from fat); HF + H1S Rodent diet: high fat (60% cal. from fat), L-methionine (7.5 gm/kg); HF + H2S Rodent diet: high fat (60% cal. from fat), 6 nutrients (15 g/kg choline chloride, 15 g/kg betaine (anhydrous), 15 mg/kg folic acid, 1.5 mg/kg vitamin B12, 7.5 g/kg L-methionine, and 150 mg/kg zinc from ZnSO_4_·7H_2_O).

**Table 2:** Summary of DMRs by HF and H2S diets.

	HF vs. NF			H2S vs. NF		
	Hypo-Met DMR	Hyper-Met DMR	Total	Hypo-Met DMR	Hyper-Met DMR	Total
Acsl1	21 (100%)	0 (0%)	21	0 (0%)	2 (100%)	2
Akr1a1	0 (0%)	4 (100%)	4	0 (0%)	30 (100%)	30
Hadh	0 (0%)	1 (100%)	1	17 (68%)	8 (32%)	25
Atg2b	4 (33.33%)	8 (66.67%)	12	6 (26.09%)	17 (73.91%)	23
Atp5h	0 (0%)	144 (100%)	144	0 (0%)	20 (100%)	20
Cox7a2l	2 (7.69%)	24 (92.31%)	26	0 (0%)	0 (0%)	0
Ndufb9	12 (27.27%)	32 (72.72%)	44	0 (0%)	72 (100%)	72
Ndufs4	0 (0%)	10 (100%)	10	0 (0%)	3 (100%)	3
Total	39 (14.89%)	223 (85.11%)	262	23 (13.14%)	152 (86.86%)	175

**Table 3:** DMRs induced by HF-diet and recovered by the H2S diet.

	# of HF-Induced DMRs	Recovered DMRs		Un-Recovered DMRs		Additional DMRsInduced by H2S	*p* Value HF vs. H2S (Fisher Exact Test)
		#	% of HF-Induced	#	% of HF-Induced		
Acsl1	21	21	100	0	0	2	0
Akr1a1	4	1	25	3	75	27	0
Hadh	1	0	0	1	100	24	0
Atg2b	12	12	100	0	0	23	0.054
Atp5h	144	124	99.31	1	0.69	19	0
Cox7a2l	26	24	100	0	0	0	0
Ndufb9	44	41	93.18	3	6.82	69	0
Ndufs4	10	10	100	0	0	3	0.051
Total	262	233	96.95	8	3.05	167	--

#: Number(s).

## Data Availability

WGBS data is accessible at the BioProject database (BioProject ID: PRJNA777555).

## Supplementary Materials

The following supporting information can be downloaded at: https://www.mdpi.com/article/10.3390/nu14122545/s1, Table S1: Details of DMRs by NF, HF and H2S diets.

## References

[1] Younossi Z., Tacke F., Arrese M., Sharma B.C., Mostafa I., Bugianesi E., Wong V.W.-S., Yilmaz Y., George J., Fan J. (2019). Global Perspectives on Nonalcoholic Fatty Liver Disease and Nonalcoholic Steatohepatitis. Hepatology.

[2] Younossi Z., Anstee Q.M., Marietti M., Hardy T., Henry L., Eslam M., George J., Bugianesi E. (2017). Global burden of NAFLD and NASH: Trends, predictions, risk factors and prevention. Nat. Rev. Gastroenterol. Hepatol..

[3] Ge X., Zheng L., Wang M., Du Y., Jiang J. (2020). Prevalence trends in non-alcoholic fatty liver disease at the global, regional and national levels, 1990–2017: A population-based observational study. BMJ Open.

[4] Clark J.M. (2006). The epidemiology of nonalcoholic fatty liver disease in adults. J. Clin. Gastroenterol..

[5] Le Clair C., Abbi T., Sandhu H., Tappia P.S. (2009). Impact of maternal undernutrition on diabetes and cardiovascular disease risk in adult offspring. Can. J. Physiol. Pharmacol..

[6] Leddy M.A., Power M.L., Schulkin J. (2008). The impact of maternal obesity on maternal and fetal health. Rev. Obstet. Gynecol..

[7] Metges C.C. (2009). Early Nutrition and Later Obesity: Animal Models Provide Insights into Mechanisms. Adv. Exp. Med. Biol..

[8] Muhlhausler B.S., Ong Z.Y. (2011). The fetal origins of obesity: Early origins of altered food intake. Endocr. Metab. Immune Disord.-Drug Targets.

[9] Rooney K., Ozanne S. (2011). Maternal over-nutrition and offspring obesity predisposition: Targets for preventative interventions. Int. J. Obes..

[10] Simar D., Chen H., Lambert K., Mercier J., Morris M. (2012). Interaction between maternal obesity and post-natal over-nutrition on skeletal muscle metabolism. Nutr. Metab. Cardiovasc. Dis..

[11] Williams L., Seki Y., Vuguin P.M., Charron M.J. (2013). Animal models of in utero exposure to a high fat diet: A review. Biochim. Biophys. Acta (BBA)-Mol. Basis Dis..

[12] Yang Z., Huffman S.L. (2012). Nutrition in pregnancy and early childhood and associations with obesity in developing countries. Matern. Child Nutr..

[13] Hivert M.-F., Rifas-Shiman S.L., Gillman M.W., Oken E. (2016). Greater early and mid-pregnancy gestational weight gains are associated with excess adiposity in mid-childhood. Obesity.

[14] Pietrobelli A., Agosti M., The MeNu Group (2017). Nutrition in the First 1000 Days: Ten Practices to Minimize Obesity Emerging from Published Science. Int. J. Environ. Res. Public Health.

[15] Chmurzynska A., Stachowiak M., Gawecki J., Pruszynska-Oszmalek E., Tubacka M. (2011). Protein and folic acid content in the maternal diet determine lipid metabolism and response to high-fat feeding in rat progeny in an age-dependent manner. Genes Nutr..

[16] Kumar K.A., Lalitha A., Pavithra D., Padmavathi I.J., Ganeshan M., Rao K.R., Venu L., Balakrishna N., Shanker N.H., Reddy S.U. (2013). Maternal dietary folate and/or vitamin B12 restrictions alter body composition (adiposity) and lipid metabolism in Wistar rat offspring. J. Nutr. Biochem..

[17] McKay J., Xie L., Manus C., Langie S., Maxwell R.J., Ford D., Mathers J.C. (2014). Metabolic effects of a high-fat diet post-weaning after low maternal dietary folate during pregnancy and lactation. Mol. Nutr. Food Res..

[18] Nathanielsz P.W., Yan J., Green R., Nijland M., Miller J.W., Wu G., McDonald T.J., Caudill M.A. (2015). Maternal obesity disrupts the methionine cycle in baboon pregnancy. Physiol. Rep..

[19] Cordero P., Gomez-Uriz A.M., Campion J., Milagro F.I., Martinez J.A. (2012). Dietary supplementation with methyl donors reduces fatty liver and modifies the fatty acid synthase DNA methylation profile in rats fed an obesogenic diet. Genes Nutr..

[20] Cordero P., Milagro F.I., Campion J., Martinez J.A. (2013). Maternal Methyl Donors Supplementation during Lactation Prevents the Hyperhomocysteinemia Induced by a High-Fat-Sucrose Intake by Dams. Int. J. Mol. Sci..

[21] Peng H., Xu H., Wu J., Li J., Zhou Y., Ding Z., Siwko S.K., Yuan X., Schalinske K.L., Alpini G. (2021). Maternal high-fat diet disrupted one-carbon metabolism in offspring, contributing to nonalcoholic fatty liver disease. Liver Int..

[22] Rakhshandehroo M., Knoch B., Müller M., Kersten S. (2010). Peroxisome Proliferator-Activated Receptor Alpha Target Genes. PPAR Res..

[23] Nohr E.A., Vaeth M., Baker J., Sørensen T.I., Olsen J., Rasmussen K.M. (2009). Pregnancy outcomes related to gestational weight gain in women defined by their body mass index, parity, height, and smoking status. Am. J. Clin. Nutr..

[24] Rasmussen K.M., Catalano P.M., Yaktine A.L. (2009). New guidelines for weight gain during pregnancy: What obstetrician/gynecologists should know. Curr. Opin. Obstet. Gynecol..

[25] Huang Y., He Y., Sun X., He Y., Li Y., Sun C. (2014). Maternal High Folic Acid Supplement Promotes Glucose Intolerance and Insulin Resistance in Male Mouse Offspring Fed a High-Fat Diet. Int. J. Mol. Sci..

[26] Xie K., Fu Z., Li H., Gu X., Cai Z., Xu P., Cui X., You L., Wang X., Zhu L. (2018). High folate intake contributes to the risk of large for gestational age birth and obesity in male offspring. J. Cell. Physiol..

[27] Wu L., Zhou X., Li T., He J., Huang L., Ouyang Z., He L., Wei T., He Q. (2017). Improved Sp1 and Betaine Homocysteine-S-Methyltransferase Expression and Homocysteine Clearance Are Involved in the Effects of Zinc on Oxidative Stress in High-Fat-Diet-Pretreated Mice. Biol. Trace Element Res..

[28] Xu L., Huang D., Hu Q., Wu J., Wang Y., Feng J. (2015). Betaine alleviates hepatic lipid accumulation via enhancing hepatic lipid export and fatty acid oxidation in rats fed with a high-fat diet. Br. J. Nutr..

[29] Bakir M.B., Salama M.A., Refaat R., Ali M.A., Khalifa E.A., Kamel M. (2019). Evaluating the therapeutic potential of one-carbon donors in nonalcoholic fatty liver disease. Eur. J. Pharmacol..

[30] Bouzidi A., Magnifico M.C., Paiardini A., Macone A., Boumis G., Giardina G., Rinaldo S., Liberati F.R., Lauro C., Limatola C. (2020). Cytosolic serine hydroxymethyltransferase controls lung adenocarcinoma cells migratory ability by modulating AMP kinase activity. Cell Death Dis..

[31] Dahlhoff C., Worsch S., Sailer M., Hummel B.A., Fiamoncini J., Uebel K., Obeid R., Scherling C., Geisel J., Bader B.L. (2014). Methyl-donor supplementation in obese mice prevents the progression of NAFLD, activates AMPK and decreases acyl-carnitine levels. Mol. Metab..

[32] Kuznetsov J.N., Leclerc G.J., Leclerc G.M., Barredo J.C. (2011). AMPK and Akt Determine Apoptotic Cell Death following Perturbations of One-Carbon Metabolism by Regulating ER Stress in Acute Lymphoblastic Leukemia. Mol. Cancer Ther..

[33] Papadopoli D.J., Ma E.H., Roy D., Russo M., Bridon G., Avizonis D., Jones R.G., St-Pierre J. (2020). Methotrexate elicits pro-respiratory and anti-growth effects by promoting AMPK signaling. Sci. Rep..

[34] Askarpour M., Hadi A., Miraghajani M., Symonds M.E., Sheikhi A., Ghaedi E. (2019). Beneficial effects of l-carnitine supplementation for weight management in overweight and obese adults: An updated systematic review and dose-response meta-analysis of randomized controlled trials. Pharmacol. Res..

[35] Del Vecchio F.B., Coswig V.S., Galliano L.M. (2016). Comment on ‘The effect of (l-)carnitine on weight loss in adults: A systematic review and meta-analysis of randomized controlled trials’. Obes. Rev..

[36] Pooyandjoo M., Nouhi M., Shab-Bidar S., Djafarian K., Olyaeemanesh A. (2016). The effect of (L-)carnitine on weight loss in adults: A systematic review and meta-analysis of randomized controlled trials. Obes. Rev..

[37] McCann M., De la Rosa M.G., Rosania G., Stringer K. (2021). L-Carnitine and Acylcarnitines: Mitochondrial Biomarkers for Precision Medicine. Metabolites.

[38] Pogribny I.P., Tryndyak V.P., Bagnyukova T.V., Melnyk S., Montgomery B., Ross S.A., Latendresse J.R., Rusyn I., Beland F.A. (2009). Hepatic epigenetic phenotype predetermines individual susceptibility to hepatic steatosis in mice fed a lipogenic methyl-deficient diet. J. Hepatol..

[39] Moody L., Wang H., Jung P.M., Chen H., Pan Y.-X. (2019). Maternal and Post-Weaning High-Fat Diets Produce Distinct DNA Methylation Patterns in Hepatic Metabolic Pathways within Specific Genomic Contexts. Int. J. Mol. Sci..

[40] Smith T., Sloboda D.M., Saffery R., Joo E., Vickers M.H. (2013). Maternal nutritional history modulates the hepatic IGF–IGFBP axis in adult male rat offspring. Endocrine.

[41] Wankhade U., Zhong Y., Kang P., Alfaro M., Chintapalli S.V., Thakali K.M., Shankar K. (2017). Enhanced offspring predisposition to steatohepatitis with maternal high-fat diet is associated with epigenetic and microbiome alterations. PLoS ONE.

[42] Zhang Q., Xiao X., Zheng J., Li M., Yu M., Ping F., Wang T., Wang X. (2019). A Maternal High-Fat Diet Induces DNA Methylation Changes That Contribute to Glucose Intolerance in Offspring. Front. Endocrinol..

[43] Ge Z.-J., Luo S.-M., Lin F., Liang Q.-X., Huang L., Wei Y.-C., Hou Y., Han Z.-M., Schatten H., Sun Q.-Y. (2014). DNA Methylation in Oocytes and Liver of Female Mice and Their Offspring: Effects of High-Fat-Diet–Induced Obesity. Environ. Health Perspect..

[44] Pirola C.J., Gianotti T.F., Burgueño A.L., Rey-Funes M., Loidl C.F., Mallardi P., Martino J.S., Castaño G.O., Sookoian S. (2012). Epigenetic modification of liver mitochondrial DNA is associated with histological severity of nonalcoholic fatty liver disease. Gut.

[45] Schwenk R.W., Jonas W., Ernst S.B., Kammel A., Jähnert M., Schürmann A. (2013). Diet-dependent Alterations of Hepatic Scd1 Expression are Accompanied by Differences in Promoter Methylation. Horm. Metab. Res..

[46] Sookoian S., Rosselli M.S., Gemma C., Burgueño A.L., Gianotti T.F., Castaño G.O., Pirola C.J. (2010). Epigenetic regulation of insulin resistance in nonalcoholic fatty liver disease: Impact of liver methylation of the peroxisome proliferator-activated receptor gamma coactivator 1alpha promoter. Hepatology.

